# Remotely sensed terrestrial open water evaporation

**DOI:** 10.1038/s41598-023-34921-2

**Published:** 2023-05-20

**Authors:** Joshua B. Fisher, Matthew B. Dohlen, Gregory H. Halverson, Jacob W. Collison, Christopher Pearson, Justin L. Huntington

**Affiliations:** 1 Schmid College of Science and Technology, Chapman University, 1 University Drive, Orange, CA 92866 USA; 2 Joint Institute for Regional Earth System Science and Engineering, University of California, Los Angeles, 607 Charles E Young Drive East, Los Angeles, CA 90095 USA; 3 Jet Propulsion Laboratory, California Institute of Technology, 4800 Oak Grove Drive, Pasadena, CA 91109 USA; 4 Department of Civil Engineering, University of New Mexico, 1 University of New Mexico, Albuquerque, NM 87131 USA; 5 Division of Hydrologic Sciences, Desert Research Institute, 2215 Raggio Parkway, Reno, NV 89512 USA

**Keywords:** Environmental sciences, Hydrology

## Abstract

Terrestrial open water evaporation is difficult to measure both in situ and remotely yet is critical for understanding changes in reservoirs, lakes, and inland seas from human management and climatically altered hydrological cycling. Multiple satellite missions and data systems (e.g., ECOSTRESS, OpenET) now operationally produce evapotranspiration (ET), but the open water evaporation data produced over millions of water bodies are algorithmically produced differently than the main ET data and are often overlooked in evaluation. Here, we evaluated the open water evaporation algorithm, AquaSEBS, used by ECOSTRESS and OpenET against 19 in situ open water evaporation sites from around the world using MODIS and Landsat data, making this one of the largest open water evaporation validations to date. Overall, our remotely sensed open water evaporation retrieval captured some variability and magnitude in the in situ data when controlling for high wind events (instantaneous: r^2^ = 0.71; bias = 13% of mean; RMSE = 38% of mean). Much of the instantaneous uncertainty was due to high wind events (*u* > mean daily 7.5 m·s^−1^) when the open water evaporation process shifts from radiatively-controlled to atmospherically-controlled; not accounting for high wind events decreases instantaneous accuracy significantly (r^2^ = 0.47; bias = 36% of mean; RMSE = 62% of mean). However, this sensitivity minimizes with temporal integration (e.g., daily RMSE = 1.2–1.5 mm·day^−1^). To benchmark AquaSEBS, we ran a suite of 11 machine learning models, but found that they did not significantly improve on the process-based formulation of AquaSEBS suggesting that the remaining error is from a combination of the in situ evaporation measurements, forcing data, and/or scaling mismatch; the machine learning models were able to predict error well in and of itself (r^2^ = 0.74). Our results provide confidence in the remotely sensed open water evaporation data, though not without uncertainty, and a foundation by which current and future missions may build such operational data.

## Introduction

Knowledge of terrestrial open water evaporation is necessary in understanding how and why changes in reservoirs, lakes, and inland seas occur, and how to manage these changes^1,2^. As climate extremes and human demand on freshwater sources increase, both society and natural ecosystems are impacted by shrinking reservoirs, lakes, and inland seas^3–8^. Two causes can shrink all these water bodies for a given known water height and surface discharge or human abstraction: (1) surface evaporation; and, (2) leakage^9,10^. Evaporation and leakage are very difficult to estimate and monitor accurately, yet only evaporation can change significantly from day to day, diurnally, and seasonally^11^. Half the water loss may be from evaporation alone, and could be roughly equal to human abstraction, which in summation may be approximately equal to total inputs if water levels stay constant; a shift in any of those can alter the equilibrium balance^12,13^. Understanding surface evaporation rates can help close the water balance equation and inform decision-makers on how to manage abstraction and movement of water from these bodies^10,14–16^. For instance, water managers may have multiple interconnected reservoirs in their purview, and may be able to move water from one reservoir to the next if that can enable them to reduce evaporative loss^17–20^.

Measuring open water evaporation is challenging for two primary reasons: (1) instrumentation; and, (2) representation^1,16^. It is difficult to set up and maintain in situ evaporation equipment in the middle of an unstable water body^21–24^. Eddy covariance assumptions about stability and fetch are often ill-constrained^25^, Bowen ratio approaches lose temporal fidelity^26^, and bulk aerodynamic or mass transfer methods are sensitive to user-(mis)calibration^27^. Further, most methods are indirect estimates of evaporation. Related, representation of point measurements to the larger water body is often error-prone as evaporation rates vary widely across the body depending on underlying bathymetry—and associated radiative storage and cycling—and exposure to varying wind and humidity especially from on-shore^28–30^.

Remote sensing has the potential to overcome the spatial representation problem, especially given high spatial resolution measurements of surface temperature^31,32^. Such resolution not only can identify spatial variability in evaporation, but also dynamically changing surface area related to water height and subsequent volumetric water loss^33^. In conjunction with high frequency in situ measurements, this combination can be a powerful pair both to produce a fused spatiotemporal capability, as well as provide calibration of the satellite data^9,34^. Still, there are challenges with remotely based evaporation estimates both in terms of retrieval mathematical formulation and physical process assumptions, as well as available data to drive those models^35–37^. Numerous models have been developed to estimate open water evaporation^38–46^.

Here, we are motivated by NASA’s ECOSTRESS mission, which now operationally produces open water evaporation data over millions of water bodies^47^ using the AquaSEBS approach^45^ in the global evapotranspiration (ET) product (L3_ET_PT-JPL)^32^. Initially in data Collection 1, ECOSTRESS masked out water bodies because the open water evaporation algorithm had not been evaluated. The analysis that went into this paper provided confidence in the open water evaporation data^48^, which are now un-masked in Collection 2. Because back-processing of the ECOSTRESS Collection 2 data takes a long time (e.g., > 1 year) and were consequently unavailable, we evaluated the ECOSTRESS open water evaporation algorithm using MODIS and Landsat data here. Moreover, PT-JPL with AquaSEBS is a core model in the OpenET system, including the open water evaporation component, which is linked to the ECOSTRESS implementation^49^. Our primary objective here is to establish the dataset and approach, and provide a first initial evaluation (Stage 1) of the AquaSEBS open water evaporation model as implemented in both ECOSTRESS and OpenET. We compiled in situ data from 19 sites from around the world, making this paper one of the largest evaluations of remotely sensed terrestrial open water evaporation to date. We also benchmarked AquaSEBS against a suite of 11 machine learning approaches to determine what the best accuracy is for a calibrated and optimized statistical model. This paper establishes a foundation on which subsequent analyses may be done with ECOSTRESS and OpenET data and provides an important reference for science investigations that use the open water evaporation data.

## Methods

### Data: in situ

**Figure 1:**
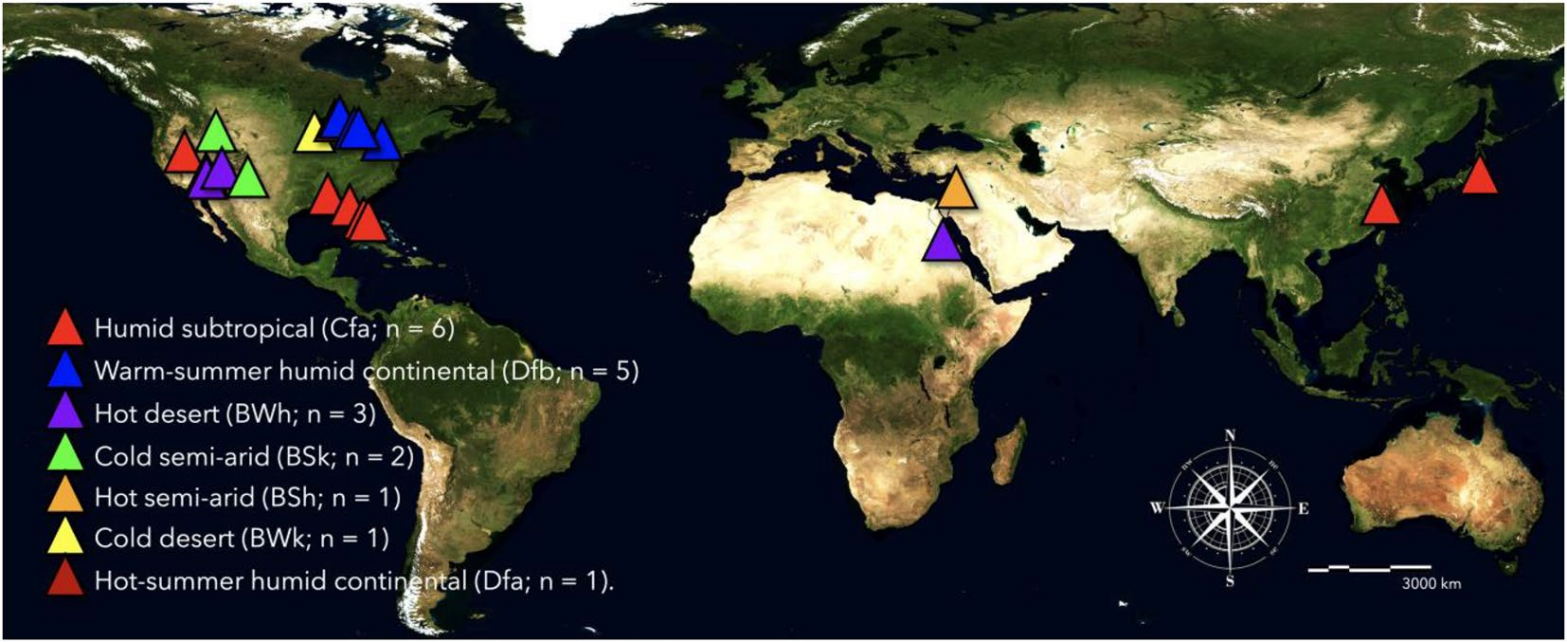
In situ terrestrial open water evaporation data from 19 sites around the world were used to validate the remote sensing data. QGIS version 3.18 and Microsoft PowerPoint version 16.72 were used to add map elements to the figures.

In situ measurements and estimates of open water evaporation and ancillary data were collected from sample size (n) 19 sites from around the world (Fig. 1). The sites included reservoirs and lakes of varying sizes and seven different Köppen-Geiger climate zones^50^: humid subtropical (Cfa; n = 6), warm-summer humid continental (Dfb; n = 5), hot desert (BWh; n = 3), cold semi-arid (BSk; n = 2), hot semi-arid (BSh; n = 1), cold desert (BWk; n = 1), and hot-summer humid continental (Dfa; n = 1). Data were obtained from the Great Lakes Evaporation Network (GLEN) (superiorwatersheds.org/GLEN)^21,29^, the US Bureau of Reclamation’s Open Water Evaporation Network (OWEN) (owen.dri.edu)^51^, data in Zhao and Gao^9^, as well as primary data collected by us^52^ (Table 1). Data contained within these sources (especially Zhao and Gao^9^) contain compilations of data from other studies as well^22,53–64^. Measurement techniques varied across sites including eddy covariance (n = 11), Bowen ratio energy balance (n = 5), bulk mass transfer (n = 2), and floating evaporation pan (n = 1). Ultimately, each site was handled consistently in comparisons despite differences in measurement technique and processing. Data spanned the years 1986 to 2019.

**Table 1:** List of 19 validation sites for in situ open water evaporation data.

Site	Latitude	Longitude	Climate	Method	Sample rate
American Falls	42.899761	−112.75799	Bsk	Bulk mass transfer	30 min
Calm	28.142088	−82.582044	Cfa	BREB	Monthly average
Cochiti	35.616928	−106.31541	Bsk	Floating evaporation pan	1 h
Five-O	30.42177	−85.664162	Cfa	BREB	Monthly average
Granite Island	46.7207104	−87.413556	Dfb	Eddy covariance	30 min
Kasumigaura	36.0430556	140.411667	Cfa	Eddy covariance	Monthly average
Kinneret	32.8166667	35.6	Bsh	BREB	Monthly average
Lahontan	39.4501944	−119.06874	Bwk	Bulk mass transfer	30 min
Long Point	42.56667	−80.05	Dfb	Eddy covariance	30 min
Mead	36.0834882	−114.78053	Bwh	Eddy covariance	30 min
Mohave	35.4272222	−114.64806	Bwh	Eddy covariance	30 min
Nasser	23.953539	32.877671	Bwh	BREB	Monthly average
Ross Barnett	32.43823	−90.03168	Cfa	Eddy covariance	Monthly average
Spectacle Reef	45.77581	−84.135914	Dfb	Eddy covariance	30 min
Stannard Rock	47.18361	−87.225	Dfb	Eddy covariance	30 min
Starr	27.956697	−81.588115	Cfa	BREB	Daily average
Taihu	31.382029	120.195508	Cfa	Eddy covariance	Monthly average
White Bear	45.07703	−92.98331	Dfa	Eddy covariance	Monthly average
White Shoal	45.77333	−85.13667	Dfb	Eddy covariance	30 min

The sites included reservoirs and lakes of varying sizes and seven different Köppen climate zones: humid subtropical (Cfa), warm-summer humid continental (Dfb), hot desert (BWh), cold semi-arid (BSk), hot semi-arid (BSh), cold desert (BWk), and hot-summer humid continental (Dfa). Measurement techniques varied (BREB: Bowen ratio energy balance), as well as sample rate of available data.

Data were available in different time units: (i) half-hourly (n = 9); (ii) hourly (n = 1); (iii) daily average (n = 1); and, (iv) monthly average (n = 8). Data were reported in different physical units as well. For half-hourly and hourly measurements, we converted them to match the units of the instantaneous overpass satellite data where needed, i.e., W·m^−2^. For daily and monthly data, we created daily and monthly satellite products from the instantaneous data (“Model: aquasebs water heat flux”). We did not construct new daily data for the sites with sub-daily data because of missing data. In situ data were filtered for bad quality flags as provided.

### Data: satellite: Landsat and MODIS

We produced fine spatial resolution 30 m images of water evaporation using the Landsat Analysis Ready Dataset (ARD) Surface Temperature (ST) and Surface Reflectance (SR) products^65^. This record provides a historical analog to the 70 m surface temperature from ECOSTRESS, with the limitation that the overpass time from Landsat is consistently around 10:30 AM, whereas ECOSTRESS provides sampling throughout the day. Landsat 5, 7, and 8 were used to cover the in situ record from 1986 to 2019. The Landsat ARD ST product provides resampled 30 m images of atmospherically corrected surface temperature from the 120 m Landsat 5, 60 m Landsat 7, and 100 m Landsat 8 thermal infrared instruments. Albedo was estimated by applying near-to-broadband coefficients to the SR product^66^.

To expand the temporal data volume beyond the 8–16 day revisit of the Landsat satellites, we opted to include daily Terra MODIS data (~ 10:30 AM overpass) at 1 km as well and conduct a comparison between the two data sources, from 2000 to 2019. Surface temperature was taken from the daily 1 km MOD11A1 product^67^. Albedo was taken from the 16-day, 500 m MCD43A3 product, which processes the bi-directional reflectance function over a combination of MODIS Terra and Aqua images from a 16-day repeat orbit^68^. Near surface air temperature and humidity were derived from the MOD07_L2 product following Famiglietti, Fisher^69^. Aerosol optical thickness from MOD04_L2 and cloud optical thickness from MOD06_L2 were used to derive solar radiation. Satellite data were filtered for bad quality flags and clouds. For Landsat, because the data volume was much smaller than that for MODIS, we additionally visually inspected each image manually to confirm success of the cloud filtering.

For the half-hourly and hourly data in situ data, the data point closest in time to satellite overpass was selected for comparison, accounting for differences in satellite overpass times. To capture some of the in situ fetch and to reduce any potential pixel noise, we calculated spatial aggregates of pixels at each of the in situ geographic coordinates and compared these aggregates to the single pixel overlying each site center point^32^. We note that a more sophisticated approach would be to conduct a temporally dynamic footprint-aware analysis for each site^70–73^, and adjust the corresponding pixels accordingly; the absence of this approach may reduce the goodness of fit in some instances^74^. Landsat was re-sampled to 30 m and a 5-by-5 pixels area of 150 m × 150 m was used for each site. MODIS, at a much coarser resolution, was limited in the area of extrapolation; we assessed a 3-by-3 pixels area for MODIS where possible. We screened for land intrusion through manual comparison to high resolution Google Earth RGB imagery. For each spatial aggregate, we calculated the mean, median, and interquartile range. We identified the optimal combination of spatial representation and statistical aggregate per site^32^.

### Model: AquaSEBS water heat flux

We used the AquaSEBS model to estimate the water heat flux, ${G}_{0w}$ (W·m^−2^)^45^:

1 $${T}_{n}=0.5\left({T}_{0}-{T}_{d}\right)$$

2 $$\eta =0.35+0.015{T}_{0}+0.0012{\left({T}_{n}\right)}^{2}$$

3 $$S\left(W\right)=3.3u$$

4 $$\beta =4.5+0.05{T}_{0}+\left(\eta +0.47\right)\times S\left(W\right)$$

5 $${T}_{e}={T}_{d}+\frac{{R}_{s}}{\beta }$$

6 $${G}_{0w}=\beta \left({T}_{e}-{T}_{0}\right)$$

where ${T}_{0}$ is water surface temperature (°C); ${T}_{d}$ is near surface air dew point temperature (°C); $u$ is wind speed (m·s^−1^); and, ${R}_{s}$ is net shortwave radiation (W·m^−2^). $S\left(W\right)$ represents a wind function (m·s^−1^); $\beta$ represents a thermal exchange coefficient (W·m^−2^·°C^−1^); and, ${T}_{e}$ represents a hypothetical equilibrium temperature (°C) when the net heat flux exchange between the water surface and the atmosphere equals zero. Formulation, definitions, and nomenclature match Abdelrady, Timmermans^45^ here for consistency.

${G}_{0w}$ was used in the Priestley–Taylor^75^ equation to calculate total evaporation, $E$ (W·m^-2^):

7 $$E=\alpha \frac{\Delta }{\Delta +\gamma }\left({R}_{n}-{G}_{0w}\right)$$

where $\alpha$ is the Priestley–Taylor coefficient of 1.26 (unitless), $\Delta$ is the slope of the saturation-to-vapour pressure curve, dependent on near surface air temperature (${T}_{a}$; °C) and water vapour pressure (${e}_{a}$; kPa), $\gamma$ is the psychrometric constant (0.066 kPa·°C^−1^), and ${R}_{n}$ is net radiation (W·m^−2^).

Landsat and MODIS (and ultimately ECOSTRESS) provide ${T}_{0}$. ${T}_{d}$ can be obtained from MODIS^69^, weather stations, or reanalysis. $u$ can be obtained from weather stations or reanalysis. ${R}_{s}$ can be obtained from MODIS, weather stations, or reanalysis. For MODIS-based modeling, we used MODIS for ${T}_{d}$, ${R}_{s}$, and ${R}_{n}$ following the ECOSTRESS Collection 1 retrieval for evapotranspiration for the global product (L3_ET_PT-JPL)^32^ with temporal upscaling for daily estimates following Verma, Fisher^76^; and, $u$ from the NCEP-NCAR Reanalysis I dataset at 6-hourly, 2.5° resolution (psl.noaa.gov)^77^. Specifically, ${R}_{s}$ was retrieved using the Forest Light Environmental Simulator (FLiES)^78,79^ and Breathing Earth System Simulator (BESS)^80–82^. Downwelling shortwave radiation (${R}_{SD}$) was calculated from eight inputs: (1) solar zenith angle; (2) aerosol optical thickness at 550 nm; (3) cloud optical thickness; (4) land surface albedo; (5) cloud top height; (6) atmospheric profile type; (7) aerosol type; and, (8) cloud type^81^. Upwelling shortwave radiation (${R}_{SU}$) was calculated from broadband surface albedo, which integrates black and white sky albedo, and ${R}_{SD}$. For Landsat-based modeling, we used ${R}_{s}$, ${T}_{d}$, humidity, and $u$ from NCEP-NCAR Reanalysis I.

The remotely sensed open water evaporation was produced as instantaneous at the time of overpass. However, some of the in situ data were available only as daily sums. As such, we produced an additional daily total remotely sensed evaporation product following the ECOSTRESS Collection 1 approach for the daily PT-JPL evapotranspiration product^83^. Specifically, diurnal incoming net radiation was sinusoidally modeled based on date and latitude, and the evaporative fraction ratio between the instantaneous evaporation and net radiation was carried forward throughout the day^84^. For brevity, we refer further details of these equations to Fisher^83^ and Bisht, Venturini^84^. To compare to those sites providing only monthly sums, we averaged all daily satellite data for a given month to make respective comparisons. We note that ECOSTRESS switches to AquaSEBS when the MODIS land/water mask is water.

### Model: machine learning

We ran a suite of 11 machine learning models to determine what the best accuracy was for a given calibrated and optimized non-mechanistic model. This creates a benchmark to differentiate error between AquaSEBS and the in situ data that can be attributed to the remote sensing or the in situ data. For example, if AquaSEBS explained 50% of the variation in the in situ data, and the best machine learning model predicted 60%, then this suggests that AquaSEBS predicted most of the explainable variation. Secondarily, we also used the machine learning models to predict the error.

The models used were: (I) Ordinary Least Squares; (II) Ridge Regression; (III) LASSO; (IV) Elastic Net; (V) Multilayer Perceptron; (VI) Tensorflow Neural Network; (VII) Decision Tree; (VIII) Random Forest; (IX) Support Vector Machine; (X) K-Nearest Neighbors; and, (XI) Gradient Boosting^85–94^. We created a testing harness to train and fine tune multiple models simultaneously using Sklearn in Python^95^. Statistics on model performance and hyperparameter grid search for optimal hyperparameters were automatically saved; *k-*fold cross validation was used in conjunction with grid search^96^. Under/over fitting and convergence were tracked through loss and learning curve plots^97^. Preprocessing the data for machine learning required additional manual flagging of extended periods (> 2 weeks) of missing data. Data also required min–max scaling prior to ingestion into these models.

### Software packages

Python version 3.9.5 was used to process all data, run the machine learning models, and produce the map figures and machine learning scatterplot. Microsoft Excel version 16.72 was used to verify the statistics and improve the aesthetics of the validation scatterplots. QGIS version 3.18 was used to add map elements to the figures and Microsoft PowerPoint version 16.72 was used to add aesthetic improvements to the maps.

## Results

**Figure 2:**
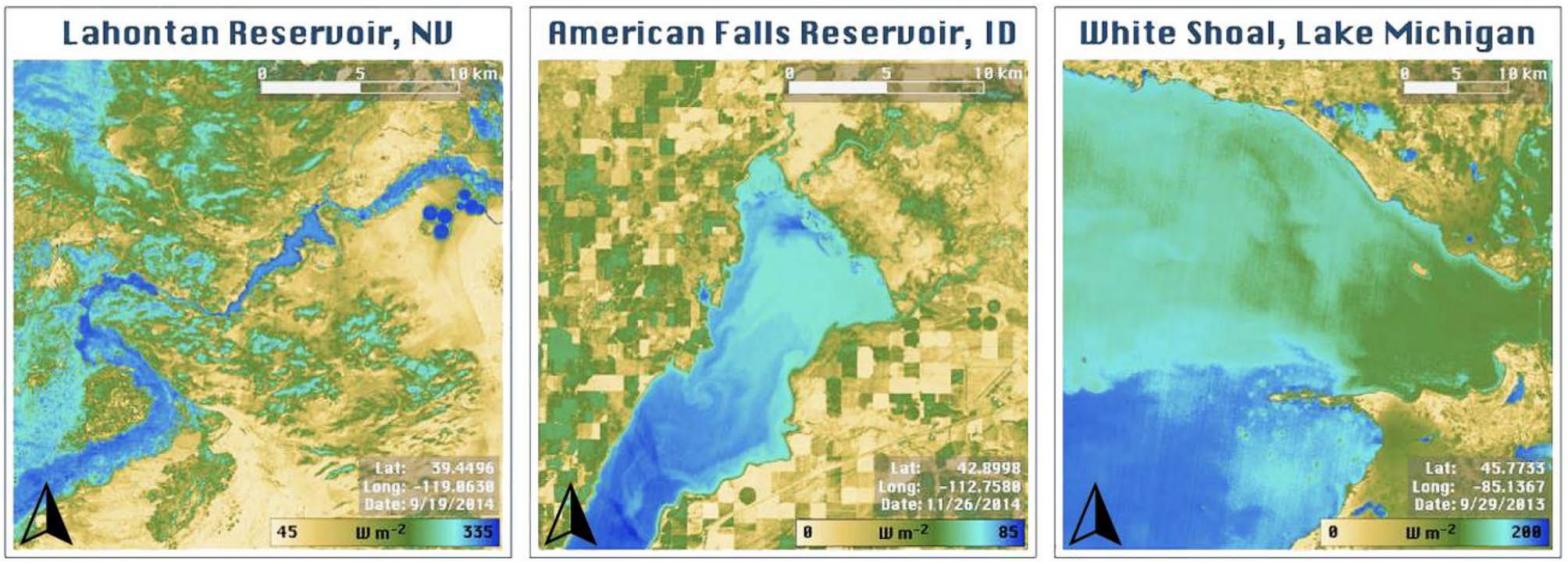
High spatial resolution (30 m Landsat) and surface temperature sensitivity of AquaSEBS reveals dynamic spatial patterns in evaporation across reservoirs and lakes. These patterns encompass near shore changes related to bathymetry, north–south or east–west gradients across the water bodies, and circulation-based patterns as heat distributes via currents. Here, three examples are shown for Lahontan Reservoir, Nevada (left); American Falls Reservoir, Idaho (middle); and, White Shoal, Lake Michigan (right). Python version 3.9.5 was used to process all data and produce the map figures. QGIS version 3.18 was used to add map elements to the figures.

We produced 30 m landscape-scale maps of open water evaporation (AquaSEBS) and land evapotranspiration (PT-JPL) from Landsat over the 19 validation sites. The high spatial resolution and surface temperature sensitivity uncovered dynamic spatial patterns in evaporation across the reservoirs and lakes (Fig. 2). These patterns encompassed near shore changes related to bathymetry, north–south/east–west gradients across the water bodies, and circulation-based patterns as heat distributes via currents. Open water evaporation was typically larger than land evapotranspiration even in irrigated or mountainous settings during late summer and fall periods.

**Figure 3:**
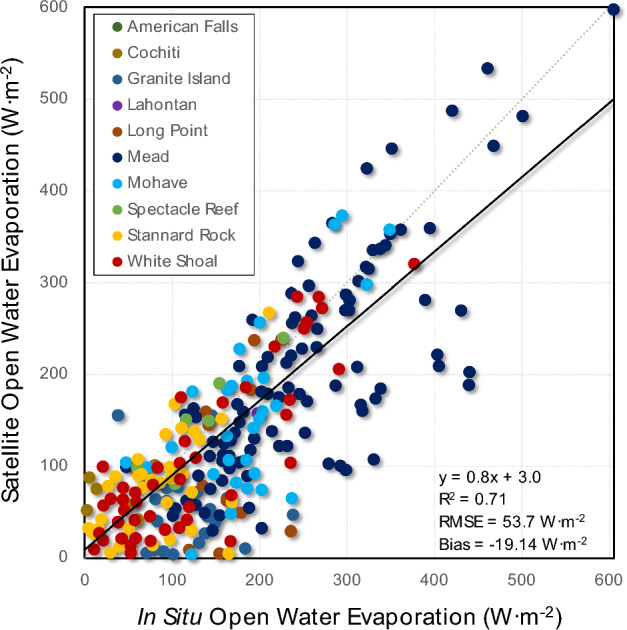
Validation of remotely sensed instantaneous open water evaporation from AquaSEBS with MODIS against in situ measurements.

The bulk of the remotely sensed data used for validation against half-hourly and hourly in situ data came from MODIS given its daily cadence. 11,016 images aligned with the available in situ data, which was filtered down to 686 cloud-free scenes. For the available in situ daily and monthly data, 52 cloud-free scenes were available. For the MODIS instantaneous data only with high wind filtering (*u* > mean daily 7.5 m·s^-1^), the remote sensing data captured the variability in the open water evaporation reasonably well (r^2^ = 0.71; RMSE = 53.7 W·m^-2^; RMSE = 38% of mean; Bias = -19.1 W·m^-2^; Bias = 13% of mean) (Fig. 3). As this is a single model (AquaSEBS) run universally across all 19 sites, this bodes well for robust extrapolation beyond the sites, which vary widely in physical and environmental characteristics.

**Figure 4:**
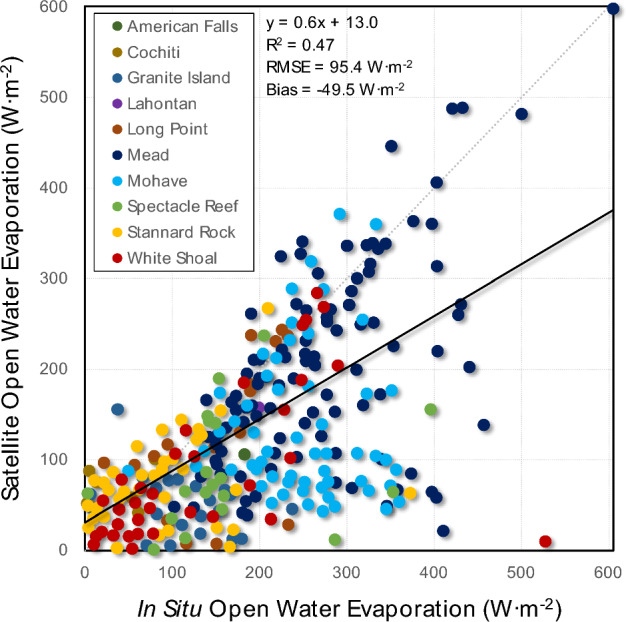
All raw unfiltered data showed high in situ instantaneous open water evaporation associated with high wind events.

**Figure 5:**
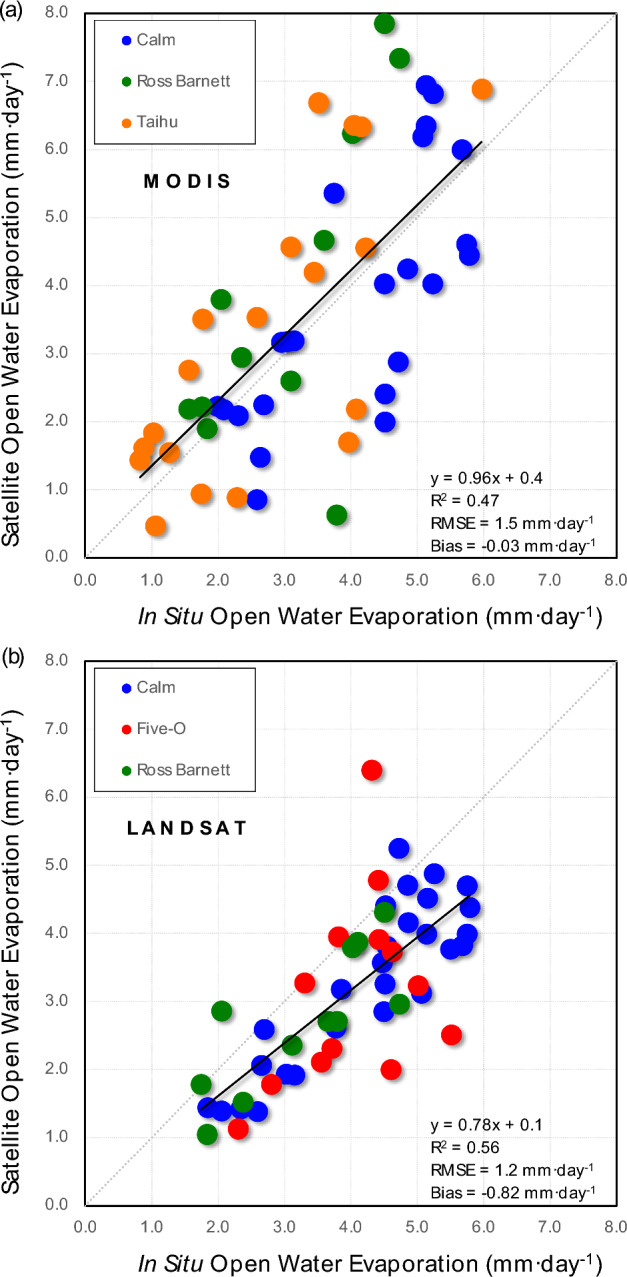
Remotely sensed daily open water evaporation data compared well with in situ data available from a limited number of sites. The daily data were less sensitive to high wind events. Results were similar between MODIS (**a**) and Landsat (**b**), though the scatter improved with the higher spatial resolution of Landsat.

However, the instantaneous results were particularly sensitive to short-term high wind events. Failure to account for these events resulted in missed high evaporation moments (r^2^ = 0.47; RMSE = 84.4 W·m^−2^; RMSE = 62% of mean; Bias = −49.5 W·m^−2^; Bias = 36% of mean) (Fig. 4). Nonetheless, the daily results are not as sensitive to these high wind events; in fact, the Bias and RMSE were remarkably small, although the scatter was still large given the small sample size (r^2^ = 0.47; RMSE = 1.5 mm·day^−1^; RMSE = 41% of mean; Bias = 0.19 mm·day^−1^; Bias = 1% of mean) (Fig. 5a).

We next asked what the change in accuracy is with increasing spatial resolution from MODIS to Landsat. Although the sample size was small and the comparison was not 1-to-1, it appeared that there may be a modest increase in correlation with Landsat; RMSE and Bias remained relatively low though larger than that of MODIS (r^2^ = 0.56; RMSE = 1.2 mm·day^−1^; RMSE = 38% of mean; Bias = −0.8 mm·day^−1^; Bias = 26% of mean) (Fig. 5b). The small sample size was sensitive to outliers; the three outlier points in the Five-O data caused a reduction in r^2^ from 0.71 to 0.56.

**Figure 6:**
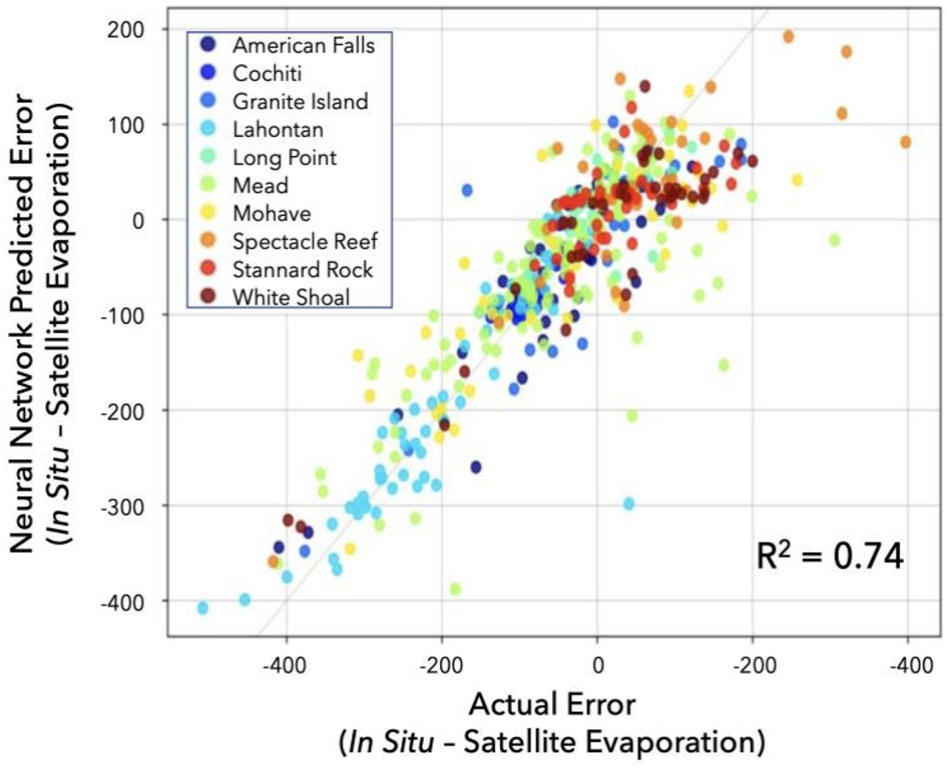
A neural network (TensorFlow) predicted error in instantaneous satellite vs. in situ open water evaporation mismatch capturing 74% of variability.

The top performing machine learning models were: (1) Multilayer Perceptron; (2) Elastic Net; (3) LASSO; (4) Ridge Regression; and, (5) TensorFlow Neural Network. However, none of the machine learning models outperformed AquaSEBS, which provides confidence for AquaSEBS and its application beyond the validation sites. Nonetheless, the machine learning models outperformed basic multiple linear regression and residual analyses in predicting AquaSEBS error (Table 2). Although error predictability varied from site to site, generally wind speed was the predominant predictor of open water evaporation error at most, but not all, sites and mostly only for the instantaneous/half-hourly/hourly data. The TensorFlow Neural Network with two hidden layers, 256 neurons at each hidden layer, dropout of 0.5 added after each layer, 0.001 regularization alpha, an Adam optimizer, ReLU for the dense layers activation function, and a loss function of mean absolute error was able to capture with reasonable speed a large amount of the variability in open water evaporation error (r^2^ = 0.74) (Fig. 6).

**Table 2:** Simple ordinary least squares linear regression analysis of ancillary predictors of error in remotely sensed open water evaporation relative to in situ measurements highlights the sensitivity to wind speed.

Variable	Coef	Std err	t	P >|t|	[0.025	0.975]
Air temperature (Celsius)	−2.00	1.54	−1.30	0.19	−5.03	1.02
Net radiation (W·m^−2^)	−0.05	0.04	−1.19	0.24	−0.14	0.03
Relative humidity (%)	−1.56	0.40	−3.92	**0.00**	−2.34	−0.77
Water vapor density (g·m^−3^)	4.69	2.61	1.80	0.07	−0.45	9.82
Wind direction (°)	0.14	0.06	2.40	**0.02**	0.03	0.26
Wind speed (m·s^−1^)	20.10	1.81	11.13	**0.00**	16.55	23.66

Significant values are in bold.

## Discussion

AquaSEBS is a relatively new model and has been used in only a few studies thus far. Abdelrady, Timmermans^45^, who developed AquaSEBS, reported RMSE’s of 20–35 W·m^−2^ and 1.5 mm·day^−1^, depending on site and measurement method; they reported a high r^2^ of 0.98 at one site though the r^2^ for their sensible heat flux was 0.70. Rodrigues, Costa^98^ and Rodrigues, Costa^37^ used AquaSEBS across multiple sites and reported RMSE’s of 0.81–1.25 mm·day^−1^ in the former and 0.03–0.58 mm·day^−1^ in the latter, with r^2^’s of 0.51–0.65 and 0.32–0.63, respectively. Our results compare similarly to all these studies (outside of the anomalous high r^2^ in Abdelrady, Timmermans^45^). Our instantaneous RMSE was a little larger than Abdelrady, Timmermans^45^ at 53.7 W·m^−2^, but our daily RMSE’s of 1.2–1.5 mm·day^−1^ compare similarly to the 1.5 mm·day^−1^ of Abdelrady, Timmermans^45^ and the high end of 1.25 mm·day^−1^ from Rodrigues, Costa^98^.

What are the sources of error in the model-data mismatch? Although we often attribute errors entirely to the model, the error is in fact a combination of multiple sources including: (1) the model; (2) the in situ data; (3) the forcing data; (4) scale mismatch; and, (5) user error^35,36^. In situ measurement of open water evaporation is challenging, as described in the Introduction; therefore, some of the model-data mismatch is due to the in situ validation data alone. Error characterization of these measurements is also challenging, and there have been numerous community efforts and controversies to make these systematic^99–103^.

Similarly, model forcing data have inherent uncertainties and errors that propagate through to the final model error^104–108^. Related, the model forcing data and final model output may be of a coarser spatial resolution than the footprint of the in situ measurements. So, the model may be “seeing” fluxes, surface, and meteorological features not captured by the in situ measurements; or, the pixels may be fine resolution, but the in situ fetch footprint is dynamic in space and time not aligned with the pixel analysis^32,70–73^. For instance, horizontally or laterally advected air, energy, and moisture can lead to both contamination in in situ measurements as well as remote sensing pixels^49,106,109,110^. For our validation, we accounted for it by a combination of: (A) placement of in situ measurements far from shore; and, (B) selection of pixels far from shore. This minimized bias from wind direction; we find no significant biases by waterbody area or shape (Table 2). However, beyond the validation, pixels at sharp wet/dry boundaries, especially in arid areas, will likely be impacted by advection^49,106,110^.

Temporal aggregation is another source of scale mismatch error, which is particularly acute when comparing instantaneous remotely sensed estimates to daily or monthly in situ data^82,111^. Such temporal mismatches, in turn, circle back to controls on the open water evaporation process and how they are represented in the model formulation. If additional model capabilities, such as machine learning approaches to predict and integrate many of these ill-constrained error sources (e.g., Fig. 6), can be combined with the process model, then there may be avenues for improving the uncertainty of remotely sensed estimates of open water evaporation.

Taken together, these challenges and limitations in both model-data mismatch and site representation present some opaqueness with understanding how accurate our remotely sensed open water evaporation data are globally. Our validation sites, while among the largest open water validate site collections to date, are not globally representative, lacking important low- and high-latitudes (and altitudes)^112,113^. Nonetheless, we chose to proceed with this collection as a critical step forward in understanding the accuracy of our data but recognize that there are more steps to be had in future analyses. On the other hand, the net gain of insight from this analysis far outweighs these limitations, making these results a significant contribution to the scientific literature.

Penman^44^ described and formulated evaporation from open water over a lifetime ago. Since then, multiple papers have described and synthesized how the controls on open water evaporation are not static but vary in space and time^1,12,114^. Still, study of terrestrial open water evaporation has been overshadowed by much more work done on evapotranspiration from land and plants^115^ or implicitly subsumed into analyses of pan evaporation^116^. Studies of ocean evaporation have been mature with low uncertainties having been reported for decades^117–121^. However, the processes controlling open water evaporation from reservoirs and lakes, while perhaps not different in name from those controlling land and plant evapotranspiration, have notably distinctive sensitivities and impacts on open water evaporation. Certainly, standard variables of radiation, humidity, wind, and air and surface temperature control both evaporation processes^122,123^. Plants and land introduce additional surface, aerodynamic, and stomatal resistances, as well as varying access to water^110,124^. But, open water introduces much deeper radiation-absorbing characteristics than do plants and land that ultimately manifest in evaporation, though not necessarily immediately or even in the same location, as heat is circulated throughout the water body^12,125^. These characteristics are, in turn, strongly determined by the physical structure of the water holding landform and associated underlying bathymetry^1^. Open water may be more sensitive to wind events than in forested ecosystems, which provide some physical structural buffering^126^. Indeed, open water evaporation may be near zero even on the hottest and driest day if there is no wind^127^. Still, evaporation formulations that incorporate wind speed are highly vulnerable to uncertainties in wind speed measurements^105^. Salinity may impact predictive capacities in both systems^45,128^. Ultimately, radiation continues to be the dominant driver of both land and open water evaporation at large space and time scales; but, the process shifts to atmospherically-controlled at short time scales^32,129–131^.

The future of remotely sensed terrestrial open water evaporation is promising with new missions emerging that increase the spatial resolution and frequency of surface temperature measurements, and operational data products including open water evaporation over millions of water bodies. The Landsat record continues to be supported with regular launches to replace aging satellites^132^. ECOSTRESS has increased the temporal resolution to 1–5 days with diurnal sampling^32^. SBG, TRISHNA, and LSTM will provide consistent, high quality, and well-calibrated surface temperature measurements every 3 days^133–135^. Hydrosat will provide the highest spatiotemporal surface temperature measurements at 50 m, daily, multiple times per day^136–138^. In a very complementary approach, radar measurements from SWOT will enable monitoring of changes in reservoir and lake water height levels, at least for large water bodies^139^. Synergies among all these missions, in conjunction with operational open water evaporation data production, can provide a step-change in our ability to estimate and monitor open water evaporation. Moreover, synergies with operational meteorological reanalyses and forecasting agencies and datasets enable diurnally integrated open water evaporation accounting from the instantaneous remote sensing measurements^49^. While reanalysis can provide fine scale temporal information, remote sensing can provide fine scale spatial measurements that can also be used to downscale the coarse reanalysis pixels^140^. Together, these tools can complement and build on foundational work done by Zhao, Li^2^ characterizing 1.42 million lakes globally, and beyond. Finally, bottom-up support of in situ monitoring networks such as GLEN^21,29^, OWEN^51^, the Global Lake Ecological Observatory Network (GLEON)^141^, the Western Reservoir Evaporation Network (WREN)^1^, and AmeriFlux/FLUXNET^142^ is necessary to provide and expand the validation and diurnal scaling as these satellite data come online.

## Conclusion

Here, we conducted the first evaluation of the AquaSEBS open water evaporation model as implemented in the ECOSTRESS mission and OpenET, applied to MODIS and Landsat data across 19 sites from around the world, making it among the largest open water validations to date. Our paper provides the foundational reference of preliminary results that provide confidence in the model and data, which enable both ECOSTRESS and OpenET to move forward with operational production and public releases of these data, and by which further research can build off. As those data begin to be produced, this evaluation should be re-visited with the new data across more sites. Moreover, further investigation is warranted to increase the sophistication from this analysis, particularly with incorporation of approaches to reduce uncertainties. These range from improving the quantification of the in situ data error and dynamic footprints to integration with machine learning techniques to predict error. There continues to be scope for improving the mechanistic formulation of the open water evaporation process within AquaSEBS with respect to environmental sensitivities and temporal dynamics for future data production collections and expanded validation sites. Synergies with upcoming missions from SBG, TRISHNA, LSTM, and Hydrosat, as well as SWOT, in conjunction with expanded and standardized in situ networks will be key to ensuring water management and analysis of changes in climate and hydrological cycling best leverage these data as such operational information becomes increasingly important into the future.

## Data Availability

The satellite datasets analyzed during the current study are available from the LP DAAC AppEEARS tool: lpdaac.usgs.gov/tools/appeears. The reanalysis data are available from: psl.noaa.gov. The in situ data were manually compiled and are available from the corresponding author on reasonable request.
